# Penile Fracture; an Emergency Managed Successfully in A Rural Setting: A Case Report

**DOI:** 10.1002/ccr3.71793

**Published:** 2026-01-02

**Authors:** Sandeep Sapkota, Amisha Adhikari, Narayan Dulal

**Affiliations:** ^1^ Department of Surgical Oncology BP Koirala Memorial Cancer Hospital Bharatpur Nepal; ^2^ Bulingtar Primary Health Care Center Nawalparasi Nepal; ^3^ Department of Urology Baratpur Hospital Bharatpur Nepal

**Keywords:** cavernosum, eggplant deformity, penile fracture, surgical repair, tunica albuginea

## Abstract

Penile fracture remains underreported in South Asia due to social taboos. It is primarily a clinical diagnosis, with ultrasonography aiding localization. Prompt surgical exploration and repair of the tunica albuginea and cavernosum are the gold standard. In resource‐limited settings, a vertical incision offers a less invasive and effective approach.

## 1 Introduction

Penile fracture is an uncommon presentation, but demands a prompt diagnosis and management. It is defined as the disruption of the tunica albuginea with rupture of the corpus cavernosum, typically occurring during vigorous sexual activity or blunt trauma to the erect penis [1]. Penile fracture is a rare entity. Careful history and examination can reveal the typical signs and symptoms of penile fracture which can help in early diagnosis. However, embarrassment and social taboo cause delay in seeking treatment which can lead to poor outcome [2]. Classically, the patient gives a history of hearing a cracking noise during sexual activity when the tunica ruptures, rapidly followed by pain, detumescence, and a substantial subcutaneous hematoma leading to an “eggplant deformity.” Although it is diagnosed clinically, ultrasound and MRI will help in the localization and assessment of the extent of injury. Surgical exploration and repair of the cavernosum and tunica albuginea is the gold standard in the management of penile fracture, thus minimizing the risk of erectile dysfunction in the future [3].

## 2 Case Presentation

**FIGURE 1:**
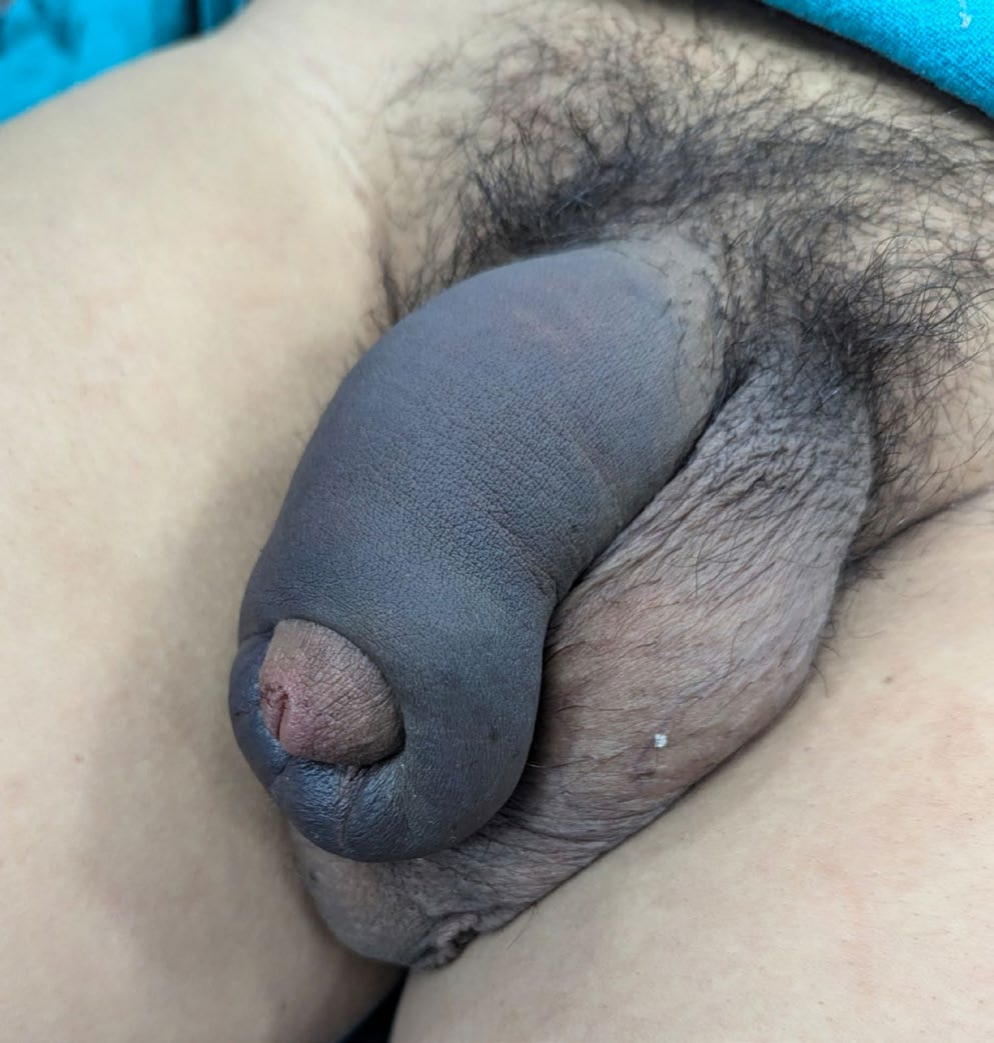
Preoperative image showing eggplant deformity.

**FIGURE 2:**
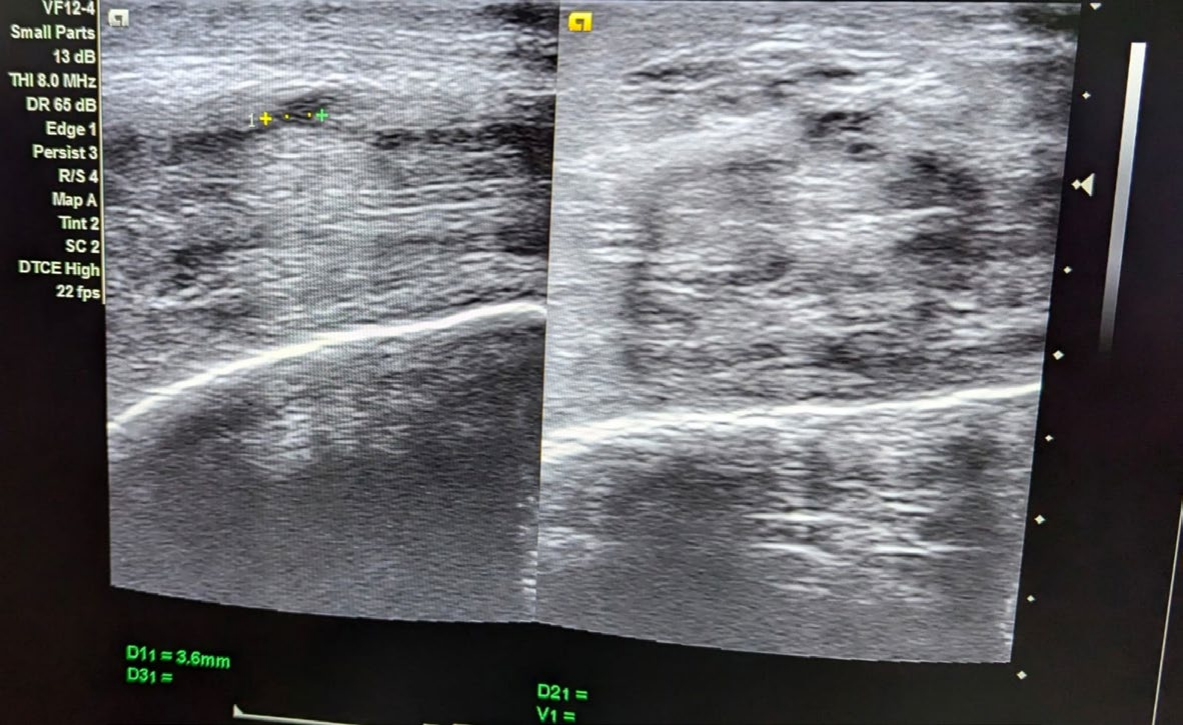
Ultrasonogram scan of penile fracture which shows the defect (right) and surrounding hematoma (left).

**FIGURE 3:**
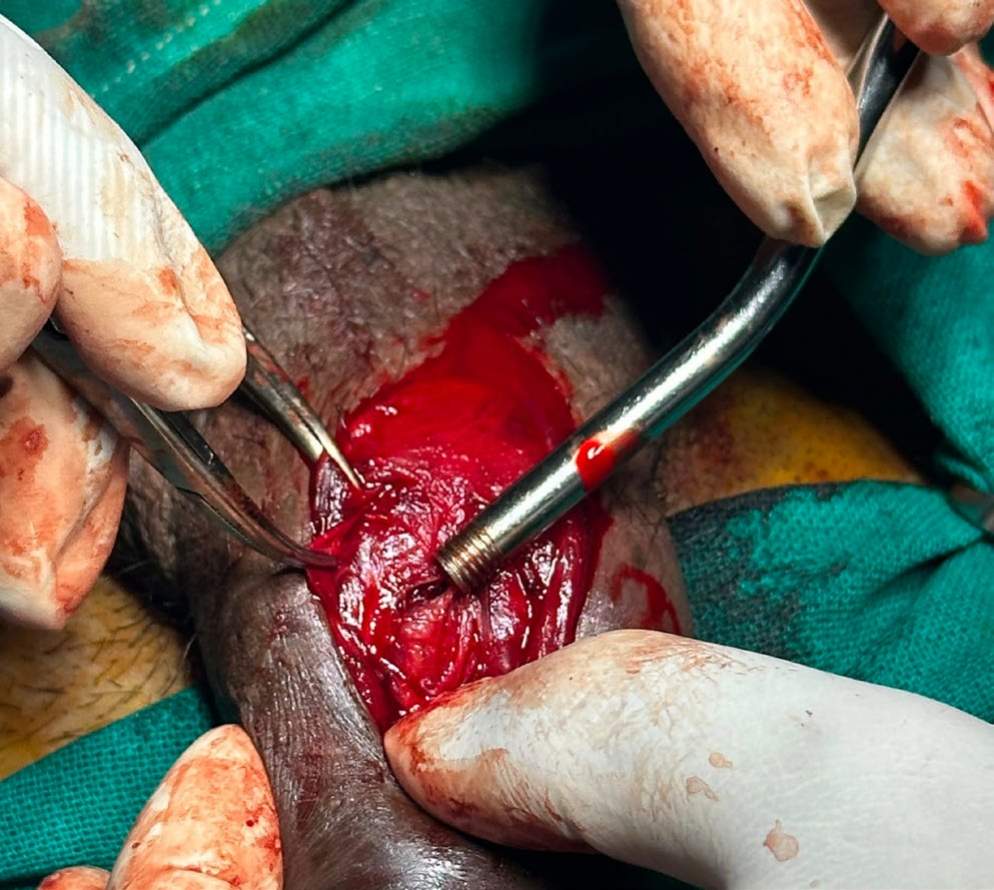
Intra operative picture showing defect in tunica albuginea.

**FIGURE 4:**
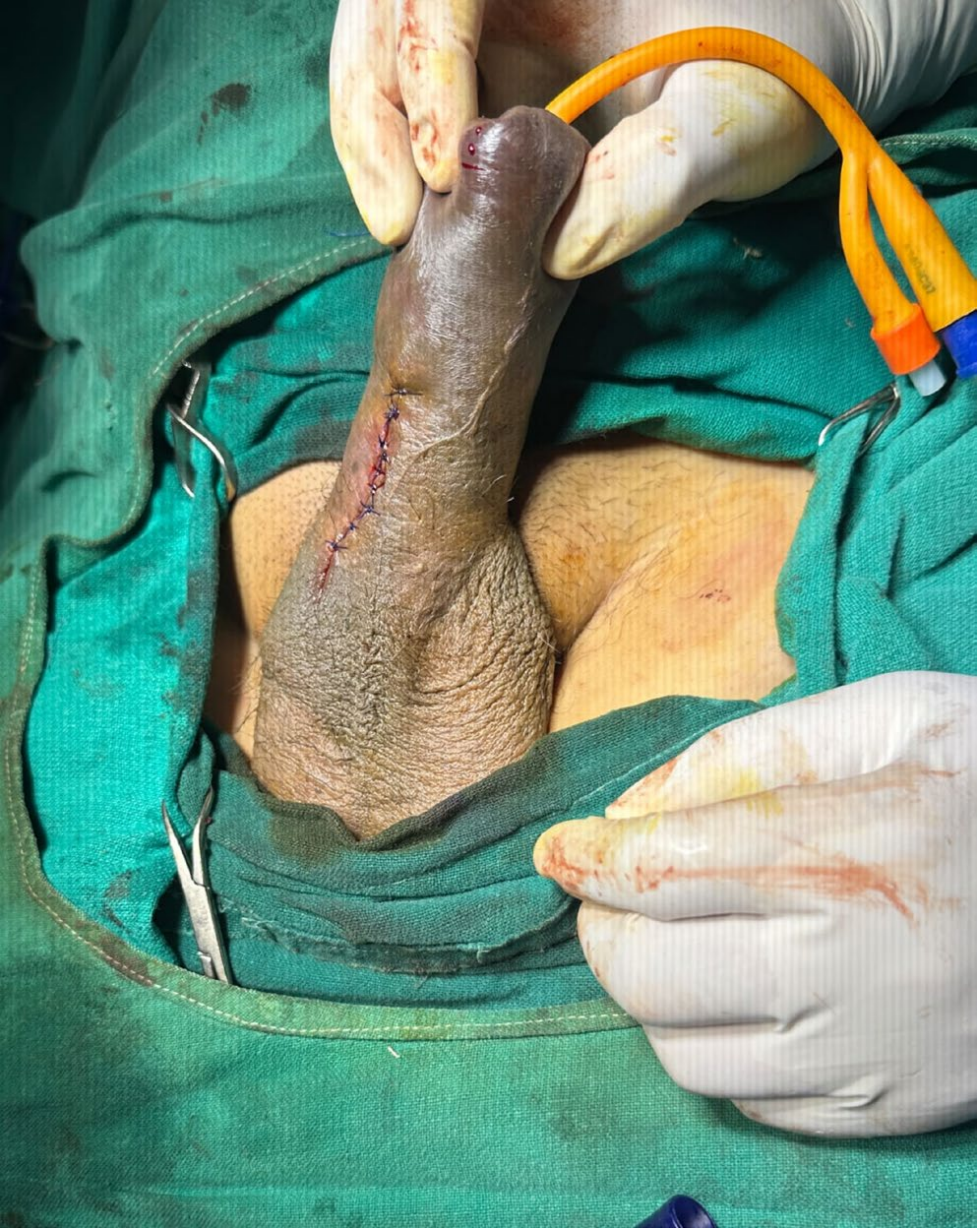
Post operative picture after local exploration and repair of penile fracture.

A 36‐year‐old, married male presented to emergency with the chief complaints of pain over the whole of the penile region for 1 day. The pain was acute in onset and the patient was hesitant to disclose the cause of the pain. After reassuring him that all the conversations we had will be highly confidential, he later revealed it to be due to pressing of erect penis against bed and there was presence of a typical “popping” sound following which he experienced pain and detumescence. On examination, penis was visibly swollen with a typical “eggplant deformity”. There was no evidence of blood in the urethral meatus which suggested that the urethra was probably not injured. After history and examination, provisional diagnosis of penile fracture was made and ultrasonogram (USG) of penis was done. On USG, breach in the tunica albuginea in the ventrolateral aspect of the right corpus cavernosum was found. There was a defect of 4.8 mm along with the surrounding echogenicity on USG. It was present 4 cm distal to the base of the penis. There was limited focal heterogeneous collection indicating hematoma which measured 5 cc along with the widespread edema of the penis. After marking the site of the defect, he was planned for operative management. There was no history of blood at urethral meatus, hematuria or voiding difficulty in the patient, so urethral injury was less likely. Moreover, there is no availability of retrograde urethrogram in our setting. Thus, the operative management was planned based on the clinical diagnosis and ultrasonography finding. He underwent complete preoperative investigations needed for the fitness of surgery which were found to be normal (Figures 1, 2, 3, 4).

During exploration, foley's catheterization was done after maintaining proper aseptic field. As the lesion was well localized by USG, a vertical incision just above the defect was given. Vertical incision is cosmetically better and less invasive as compared to a circumferential degloving incision. Thus, vertical incision was chosen in the context of resource limited setting like our hospital. A defect measuring 5 mm in the ventrolateral aspect of right corpus cavernosa with minimal hematoma was detected. The defect was closed with prolene suture.

There is a high incidence of post operative infections in urological procedures in our geographical area. So, there is a local hospital guideline advocating the use of antibiotics post operatively to minimize infection. Based on the hospital guideline for urological cases, the patient was prescribed intravenous Amoxycillin and clavulanic acid for 3 days and then switched over to oral medication. Chymotrypsin tablets were effective in reducing the diffuse penile swelling. To prevent erection during the post operative period, the most readily available drug, Diazepam was given. Regarding the use of diazepam as an aide to prevent erections during healing, this strategy is based on the work of Jallu and associates who used diazepam in conjunction with oxyphenbutazone to prevent erections in patients treated conservatively for penile fracture. There is little prospective data regarding the use of this medication, and its incorporation in management schema should not necessarily be construed as standard of care in the modern day. Patients should be counseled to abstain from sexual activity for a period of at least 6–8 weeks [4]. Foleys catheter was removed on third post operative day and patient was discharged on fourth post operative day. The patient was counseled to abstain from any sexual activities for a period of 6 weeks. The patient was kept on a regular follow up. On the 6th post operative day, dressing of the wound was done. Sutures were removed on the 10th post operative day; there were no complaints. On further follow up after 3 weeks, wound site was normal without any penile shape deformity. On his follow up after 6 weeks, the patient had no swelling or pain; he had normal urinary functions and penile erection was normal.

## 3 Differential Diagnosis

The possible differential diagnosis of a case presenting with acute onset of penile pain and swelling is as follows:

- Penile fracture
- Vascular ruptures like rupture of superficial dorsal vein, deep dorsal vein, dorsal artery of penis
- Intra cavernosal hematoma without tunica rupture
- Injury to suspensory ligament of penis
- Urethral injury without penile fracture
- Priapism, etc.
- Penile fracture with urethral injury.
  Penile fracture is a clinical diagnosis and usually presents with sudden crack sound, immediate loss of erection, and typical eggplant deformity. Vascular ruptures present with swelling without immediate detumescence

## 4 Conclusion and Results

On follow up, dressing of the wound was done. The wound healed completely on the 10th post operative day, so the sutures were removed after dressing of the wound. Pain and swelling had subsided and there was no deformity. His urinary function was normal. On his subsequent follow up after 6 weeks, he gave a history of erection. Hence, timely diagnosis and surgical management of penile fracture can result in a good outcome even in a resource limited setting like rural Nepal. Our case report has helped us to come to the following conclusions:

- Penile fracture is a true urological emergency that requires prompt diagnosis and early surgical repair to ensure optimal outcomes.
- Ultrasonography serves as a valuable tool for localizing injury in corpora cavernosa in rural settings.
- A vertical incision can be a less invasive, cosmetically better, and practical alternative to the traditional circumferential degloving approach, particularly when the lesion is well localized using ultrasonography.
- Comparison with recent literature, including cases complicated by urethral rupture, highlights the need for larger, multi‐institutional studies to validate these observations.

## 5 Discussion

Penile fractures are a urological emergency which occurs most commonly in middle aged men between 30 and 50 years of age. The reported yearly overall incidence in the United States is 1 case per 175,000 men [1]. Potential outcomes of penile fracture include erectile dysfunction, penile curvature, and urethral injury. Treatment is emergent surgical repair [4]. A large number of studies have shown that the etiology of penile fracture varies based on geographical region, sexual behavior, marital status, and culture. Sexual intercourse, masturbation, forced penile manipulation, and rolling over in bed onto the erect penis are considered common causes of this fracture [5] In a study by Moslemi, habitual manual trauma was the most common cause (56%), sexual intercourse was associated with only 30% of cases. Interestingly, their case series shows seasonal variation in penile fracture cases where the highest number of cases were seen in the summer followed by spring [6]. Penile fracture probably still remains underreported in the context of South Asian countries especially in India and Nepal.

Penile fracture is a traumatic rupture of the corpus cavernosum. Sudden blunt trauma or abrupt lateral bending of the penis in an erect state can break the markedly thinned and stiff tunica albuginea, resulting in a fractured penis. Urethral trauma is more common when both corpora cavernosa are injured [7].

Penile fracture is a clinical diagnosis; its typical history and characteristic eggplant deformity guide us towards the correct diagnosis. The role of ultrasonogram in diagnosis and finding the exact site of penile fracture cannot be undermined, especially in cases of trauma and possible multiple site fractures. It is a cheap and noninvasive approach but can be of limited use as it is operator dependent. Ultrasonogram helps to locate the site of injury [8]. The use of penile cavernosography is debatable because false negative findings are common and there is increased risk of tissue reaction to contrast material and corporal fibrosis [7]. Another investigation that needs to be done in cases of penile fracture is retrograde urethrogram as approximately 10%–20% of cases of penile fracture are associated with penile fracture. Retrograde urethrogram should be performed in the presence of urethrorrhagia, hematuria, and urinary retention as they strongly suggest urethral injury [9].

Recent evidence for timing of operative repair reinforces the importance of immediate and early surgical repair. If a clinical presentation strongly suggests penile fracture, surgical exploration should occur as soon as possible. Delaying surgical exploration should only be considered if the diagnosis is questionable and imaging can be arranged quickly. The British Association of Urologic Surgeons recommends repair within 24 h. The initial approach to surgery includes a circumcising, degloving penile incision or a longitudinal incision over the expected area of the defect [10]. Subcoronal incision with degloving and circumcision remains the most common approach worldwide because it allows for the full inspection of the corpora cavernosa and the urethra. Penoscrotal incision also offers these benefits without mobilizing the neurovascular bundle. Transverse incision over the lesion has been gaining appeal, particularly when the lesion's exact location is identified via pre‐operative imaging. These techniques resulted in no differences in complications, that is, erectile dysfunction and self‐satisfaction with body image [11]. In the reported case we have opted for less invasive vertical incision over hematoma site after proper localization of site using ultrasonogram. A vertical incision was preferred over a circumferential degloving incision due to its simplicity, reduced morbidity, and acceptable cosmetic outcome.

Thus, the above presented case is an example of how a surgical emergency like penile fracture can be managed successfully even in a remote hospital setting with limited resources. Penile fracture is basically a clinical diagnosis and when added with ultrasonography, the defect can be localized and operative management can be commenced early, even in the absence of retrograde urethrogram if urethral injury is ruled out clinically. In a resource‐limited rural setting, longitudinal incision can give better outcomes as it is less invasive and cosmetically better.

## Author Contributions

**Sandeep Sapkota:** conceptualization, data curation, supervision, writing – original draft, writing – review and editing. **Amisha Adhikari:** conceptualization, data curation, writing – original draft, writing – review and editing. **Narayan Dulal:** data curation, supervision, writing – original draft, writing – review and editing.

## Funding

The authors have nothing to report.

## Consent

Written informed consent was obtained from the patient to publish this report in accordance with the journal's patient consent policy.

## Conflicts of Interest

The authors declare no conflicts of interest.

## Data Availability

Data available on request from the authors.
